# The Propagation of Movement Variability in Time: A Methodological Approach for Discrete Movements with Multiple Degrees of Freedom

**DOI:** 10.3389/fncom.2017.00093

**Published:** 2017-10-13

**Authors:** Melanie Krüger, Andreas Straube, Thomas Eggert

**Affiliations:** ^1^Sensorimotor Neuroscience and Ageing Research Laboratory, School of Medicine, University of Tasmania, Hobart, TAS, Australia; ^2^Department of Neurology, University Hospital Munich Großhadern, Munich, Germany; ^3^Department of Sport and Health Sciences, Technical University of Munich, Munich, Germany

**Keywords:** movement coordination, variability, canonical correlation, reaching, sensory loss

## Abstract

In recent years, theory-building in motor neuroscience and our understanding of the synergistic control of the redundant human motor system has significantly profited from the emergence of a range of different mathematical approaches to analyze the structure of movement variability. Approaches such as the Uncontrolled Manifold method or the Noise-Tolerance-Covariance decomposition method allow to detect and interpret changes in movement coordination due to e.g., learning, external task constraints or disease, by analyzing the structure of within-subject, inter-trial movement variability. Whereas, for cyclical movements (e.g., locomotion), mathematical approaches exist to investigate the propagation of movement variability in time (e.g., time series analysis), similar approaches are missing for discrete, goal-directed movements, such as reaching. Here, we propose canonical correlation analysis as a suitable method to analyze the propagation of within-subject variability across different time points during the execution of discrete movements. While similar analyses have already been applied for discrete movements with only one degree of freedom (DoF; e.g., Pearson's product-moment correlation), canonical correlation analysis allows to evaluate the coupling of inter-trial variability across different time points along the movement trajectory for multiple DoF-effector systems, such as the arm. The theoretical analysis is illustrated by empirical data from a study on reaching movements under normal and disturbed proprioception. The results show increased movement duration, decreased movement amplitude, as well as altered movement coordination under ischemia, which results in a reduced complexity of movement control. Movement endpoint variability is not increased under ischemia. This suggests that healthy adults are able to immediately and efficiently adjust the control of complex reaching movements to compensate for the loss of proprioceptive information. Further, it is shown that, by using canonical correlation analysis, alterations in movement coordination that indicate changes in the control strategy concerning the use of motor redundancy can be detected, which represents an important methodical advance in the context of neuromechanics.

## Introduction

Analyzing movement variability to gain insights into movement planning and control processes has been in the focus of researchers in the field of (computational) human motor control ever since Bernstein's (1967) famous Blacksmith example, which describes that, even in highly skilled movements with high outcome stability, execution variability can be observed across repetitions. Originating from that observation, numerous studies have been conducted to investigate changes in the amount and structure of movement variability with changing external task constraints (Gera et al., 2010; van der Steen and Bongers, 2011; Krüger et al., 2012) or under manipulated availability of sensory information during movement planning and execution (Tseng et al., 2002; Krüger et al., 2011), showing that variability in movement execution is an inherent characteristic of human performance. In the last recent years, this research has significantly profited from the emergence of a range of different mathematical approaches to analyze the structure of movement variability. Approaches such as the Uncontrolled Manifold method (Scholz and Schöner, 1999) or the Noise-Tolerance-Covariance decomposition method (Müller and Sternad, 2004) allow to detect and interpret changes in movement coordination by analyzing the structure of within-subject, inter-trial movement variability. Referring to these methods, it was shown that changes in the structure of movement variability can be related to changes in movement planning and control processes due to learning, aging, and pathology (Cirstea and Levin, 2000; Müller and Sternad, 2009; Stergiou and Decker, 2011; Krüger et al., 2013).

In that context, particularly the investigation of the time course of movement variability during movement execution has stimulated theory-building in motor neuroscience and advanced our understanding of the synergistic control of the redundant human motor system. Research on reaching and pointing movements (Domkin et al., 2002, 2005; Cohen and Sternad, 2009; Krüger et al., 2012) provided empirical support for theories of motor control which postulate that the human motor system exploits its inherent redundancy to cost-optimize movement execution, such that only variability in task-relevant dimensions is minimized, a principle referred to as “minimum intervention principle” (Todorov and Jordan, 2002; Todorov, 2004).

For time course analyses of movement variability, different time points between movement start and end, usually corresponding to either certain percent of the normalized time between movement start and end or to distinct time points in the movement, such as e.g., time point of maximum velocity, are examined. Following the definition of relevant time points between movement start and end, two principally different approaches can be followed to analyze the time course of inter-trial movement variability: first, the variance structure of the effector position at a single time point during the movement can be analyzed across many movement repetitions, and conclusions can be drawn from changes of this variance structure between different time points. As an example for this approach, a range of studies using the Uncontrolled Manifold method were able to show significant differences in the control of task-relevant and—irrelevant variability in the effector space across the time course of movement execution for, e.g., sit-to-stand, shooting, or goal-directed reaching movements (Scholz and Schöner, 1999; Scholz et al., 2000; Tseng et al., 2002). However, this approach focuses on the relations between variability in the effector and task space at one or multiple points in time, but does not allow direct conclusions about statistical coupling of effector variables across time.

In contrast to this first approach, the second approach focuses on the coupling of movement variability between different time points during movement execution. Importantly, while for cyclical movements (e.g., locomotion), mathematical approaches exist to investigate the propagation of movement variability in time (e.g., time series analysis, or Lyapunov exponent, Stergiou and Decker, 2011), similar approaches are missing for discrete, goal-directed movements with a redundant effector system, such as reaching with the arm. Here, we propose canonical correlation analysis as a suitable method to analyze the propagation of within-subject variability across different time points during the execution of discrete movements. While similar analyses have already been applied for discrete movements with only one degree of freedom (DoF; Messier and Kalaska, 1999; Richardson et al., 2011; Kuang and Gail, 2015; Eggert et al., 2016), canonical correlation analysis allows to evaluate the coupling of inter-trial variability across different time points along the movement trajectory for multiple DoF-effector systems, such as the arm.

This method will be illustrated by empirical data from a study on reaching movements under normal and disturbed proprioception. Proprioception about joint positions is an important source of information for the control of complex reaching movements (Ghez and Sainburg, 1995; Bagesteiro et al., 2006). Studies on chronically deafferented patients suffering from severe peripheral sensory neuropathy showed impaired motor control of arm movements, including slowed movement execution (Gentilucci et al., 1994; Hepp-Reymond et al., 2009), increased movement variability (Gentilucci et al., 1994; Medina et al., 2010) and deteriorated movement coordination (Sainburg et al., 1993, 1995; Ghez and Sainburg, 1995). Studies of temporary peripheral deafferentation of healthy humans showed immediate adjustment to the loss of proprioception on a behavioral (Moisello et al., 2008, applying limb immobilization) and cortical level (Björkman et al., 2004a,b, applying a local anesthetic cream; Ziemann et al., 1998b, applying an ischemic nerve block). However, these studies mainly requested the production of simple motor tasks with a limited range of kinematic DoF. Studies on the production of complex motor behavior, such as reaching movements, are rare. Here, we investigated the effect of temporary proprioceptive deafferentation, induced by an ischemic block at the upper arm level, on the time course of joint angle variability of complex arm movements.

## Methods

### Participants

Fifteen healthy volunteers (mean age ± SD: 26 ± 5 years; 8 female) participated in the study. All participants were right-hand dominant as determined by the Edinburgh Handedness Inventory (Oldfield, 1971) and had normal or corrected-to-normal vision. None of the participants had any record of neurological disorder. All participants were paid for their participation and had given written informed consent prior to participation. The experimental procedure was in accordance with the Declaration of Helsinki and was approved by the Ethics Committee of the Medical Faculty of the Ludwig-Maximilians University Munich.

### Experimental set-up

**Figure 1:**
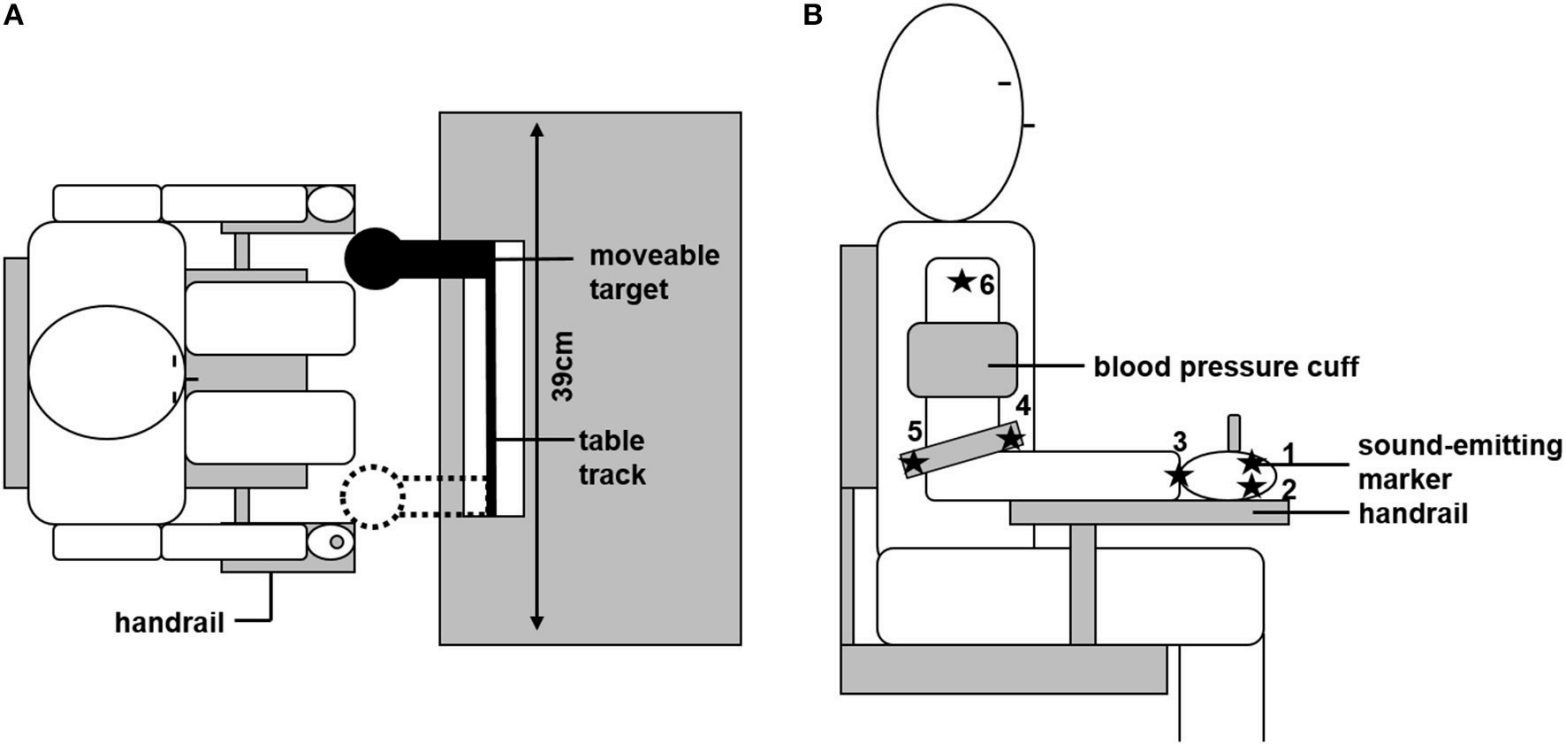
Experimental set up. **(A)** Overhead view of the experimental set-up. Sitting position was individually adjusted so that the moveable target could be reached without trunk motion. The target could be located at the two bounds of the table track. Initial starting position was defined by grasping the handrail. **(B)** Positions of the six ultrasonic sound-emitting markers and the blood pressure cuff are depicted.

Participants were seated on a chair in front of a table, with the trunk supported by a chair back. A linear table track was mounted on the table, with a spherical object (reaching target, diameter: 80 mm) attached to it. Due to its geometric properties, the reaching target constrained final hand position but not final hand orientation. The size of the target forced the participants to grasp it with the whole hand, and not just with two fingers, which is why single finger motion was not of interest in the current study. The reaching target could be freely moved horizontally (in the fronto-parallel plane) between the bounds of the table track. These bounds (distance: 39 cm) were the two positions at which the reaching target could be located. The sitting position of the participants was adjusted so that: (a) trunk movement was not necessary to reach the target, and (b) body midline was centered to the table track. To minimize within-subject inter-trial variability due to differences in the initial position, the starting position was defined by a wooden lever, attached to the right side of the chair, which had to be grasped with the dominant right hand before each trial (see Figure 1A).

Joint angle motion of the arm in its seven degrees of freedom was recorded by an ultrasonic sound-emitting system (Zebris Medical, Isny, Germany). Six sound-emitting markers were attached to the arm and hand of the participant; each marker recorded at a frequency of 33 Hz. The following marker positions were chosen and are depicted in Figure 1B: marker 1 and 2 were attached to the metacarpophalangeal joints of the index (1) and little finger (2). The third marker was at the center of the wrist. Marker 4 and 5 were attached to the medial (4) or lateral (5) end of a bracelet directly above the elbow. The sixth marker was attached at the acromion. From those marker positions, the individual length of the participant's upper arm, lower arm, and hand could be determined. Based on these lengths, a geometrical model of the arm was created, as described in more detail below (see section Data Analysis). Further, the signal of the first marker was used to trigger the opening and closing of shutter glasses (Translucent Technologies, Toronto, Canada) that were used to prevent visual online control of the movement. The first contact with the reaching target was detected by changes in the electrical resistance between the participant and the target (sampled at 1 kHz).

### Procedure and design

Participants repeatedly had to reach toward and grasp the reaching target with their dominant right hand. At the beginning of each trial, participants had to adopt the starting position (see Figure 1A). Subsequently, participants were instructed to press a button with their non-dominant hand, after which the target changed its position. After an acoustic go-signal, participants had to perform the reaching movement in a natural manner. To provoke the most natural movement behavior, participants were informed before movement recording that movement speed and reaction time were not of interest in the study. Shutter glasses occluded as soon as the participants started their movement, thus preventing visual online control of the movement. After the participants had grasped the target, the shutter glasses opened again and the participants returned to the starting position. A new trial was initiated by pressing the button again.

All participants participated in two experimental conditions in separate sessions, the order of which was counterbalanced across participants. Experimental sessions were separated by 1 to 2 days. In the first condition (“ischemia”), a customary blood pressure cuff was applied to the upper arm of the participants and inflated up to 150–160 mmHg (i.e., slightly above systolic blood pressure) to induce a transient ischemic block. Ischemic nerve block is an established experimental technique to study sensory control of movements (e.g., Glencross and Oldfield, 1975; Ziemann et al., 1998a). It is known to first affect the large, fast conducting afferent fibers, especially Ia afferents arising from the muscle spindle afferents (Fellows et al., 1993). In contrast to acute limb ischemia, a sudden decrease in limb perfusion caused by e.g., thrombosis, transient ischemic block is an incomplete block of limb perfusion caused by externally applied pressure and is non-threatening to the limb. Glencross and Oldfield (1975) showed that 20–25 min of ischemic nerve block results in complete dropout of finger sensation, and significant sensory decrease in wrist and elbow. Decrements in the exerted force were observed only after complete sensory dropout. In the current study, the duration of inflation was in a range of 20–25 min. This timeframe included 10 min of preparation to guarantee impairment in the global sensory afference, and a subsequent 10–15 min of movement recordings. Consequently, other effects of the ischemic nerve block, such as changes in producible muscle force (Björkman et al., 2004a), can be disregarded in the current set-up because of the brevity of the ischemic block. Before movement recording started, the proprioceptive impairment was tested indirectly by assessing participants' touch sensitivity with von-Frey filaments (Marstock, Schriesheim, Germany, Rolke et al., 2006). On the back of the participants' hands it was tested which of the 12 logarithmically scaled filaments (range: 0.25–512 mN) participants were at least able to perceive. On average, participants were able to perceive a minimum pressure of 0.5 mN before the application of the blood pressure cuff (i.e., mean filament number ± SD: 1.91 ± 0.53). Participants' touch sensitivity had to be reduced by at least one filament (i.e., increase by a factor 2) before the experiment was continued. On average, participants perceived a minimum of 1 mN at the start of the movement recordings (i.e., mean filament number ± SD: 3.09 ± 0.70). This procedure allowed us to be sure about the effectiveness of the ischemic block. At the same time, the duration of preparation was minimized, which was of importance to prevent unwanted side effects of the ischemic block, as for example ischemic pain. The second experimental condition (“control”) served as a control condition, executed identically but without inflated blood pressure cuff.

Two blocks of 40 trials in each block were recorded in each session (i.e., 80 trials per session). Each experimental block consisted of 20 trials of each of the two target positions, arranged in a random order to avoid predictability of the target position. Between the blocks a break of maximally 5 min was offered to avoid fatigue. Before movement recording started, participants were allowed to perform five trials to familiarize themselves with the experimental task and apparatus.

### Analysis

#### Data analysis

Data analysis was calculated using Matlab 7.9.0 (Mathworks, Natick, USA) and was in line with earlier studies by our group (Krüger et al., 2011, 2012). In a first step, the seven joint angles of the arm were computed from the marker positions using a three-segment rigid body model, and expressed as seven consecutive Cardan angles. The order of the angles was as follows: two angles for the wrist (vertical, and horizontal), two angles for the elbow (torsion, and flexion), and three angles for the shoulder (torsion, horizontal, and vertical). The zero position of the arm was defined as the arm pointing straight forward with the elbow extended and the palm facing up. Based on that, positive joint angle indicated the following directions: vertical upward, horizontal rightward, and torsion clockwise. The vector containing the seven joint angles is hereafter referred to as *arm posture*. The *position of the hand* in space (i.e., 3D) was defined by the center of the two hand markers in world fixed Cartesian coordinates. In addition, the *orientation of the hand* in space was defined in Helmholtz coordinates relative to the external world.

Temporal and spatial movement characteristics were analyzed separately for each condition, participant, target position and trial. *Overall movement duration* was defined as the time between movement initiation and movement end. To determine movement initiation, movement start was defined as the time point at which the hand velocity first exceeded 10% of its peak velocity. Movement initiation was then determined by subtracting 10% of the acceleration time (i.e., the time between movement start and reaching peak velocity) from movement start. Movement end was defined as the last sample recorded before the first contact with the reaching target, as determined by the change in electrical resistance (see section Experimental Set-Up). Subsequently, *duration of acceleration* and *duration of deceleration* were calculated. In addition, *peak velocity* was analyzed. Thus, temporal characteristics of the reaching movements will be described by four measures: (1) overall movement duration, (2) duration of acceleration, (3) duration of deceleration, and (4) peak velocity. To determine spatial characteristics of the reaching movement, movement amplitudes were determined by calculating the absolute value of the difference between the maximum and minimum joint angle separately for each of the seven joint angles. Subsequently, *mean movement amplitude* was calculated as the average movement amplitude across the seven joint angles. In addition, to evaluate the changes in the diversion from shortest trajectory between starting and end position, the *total path length* in the 7D-joint space of the arm was calculated.

Within-subject inter-trial movement variability during the time course of movement execution and at movement end was analyzed separately for each condition, participant, and target position. On that account, the full temporal resolution of the joint angle motion was reduced to 10 equidistant sampling points between movement initiation and movement end. To account for small inter-trial variations in the actual starting position of the arm and in movement duration, a correction of the joint angle trajectories was calculated as described in detail in Krüger et al. (2011). Briefly, the within-subject deviations of the joint angles from their mean were submitted to a linear regression analysis with the predictor initial arm position and movement duration (i.e., 7 + 1 = 8 continuous predictor variables). Separate regression analysis were conducted for each participant, experimental condition, target position, and for each of the 10 sampling points, thus, containing the data of 40 trials. Subsequently, the joint angle deviations from the mean that were predicted by this linear model were subtracted from the actual joint angles. After this correction, the covariance matrix of the starting position (first sample) reduced to zero and was not considered in further analytical steps. Thus, the covariance matrix of the joint angles was analyzed at nine equidistant sampling points during the movement.

Afterwards, movement variability was analyzed by means of the two approaches described in the Introduction: first, analyzing the amount of variability at the nine single sampling points during movement execution, and second, analyzing the coupling of movement variability across different sampling points. To achieve the first, the square-root of the mean within-subject variance, averaged across the seven joint angles of the arm (hereafter referred to as: *standard deviation of arm posture*), was calculated. Further, the square root of the mean within-subject variance, averaged across its three dimensions was calculated for the task variables (a) hand position (*standard deviation of hand position*) and (b) hand orientation (*standard deviation of hand orientation*). In addition, the coupling between joint angles within the arm posture at a given sampling point was analyzed by calculating a principal component analysis on the 7 × 7 covariance matrix of the arm posture. Subsequently, the variances for each of the seven eigenvalues of the covariance matrix were averaged across sampling points, and the *percentage of total variance* explained by the first two eigenvalues was calculated. A relative increase of this percentage is closely related to a relative decrease of the number of kinematic DoF with respect to the mechanical DoF.

To achieve the second, the coupling between the arm posture at a given sampling point and the final arm posture was examined. On that account, the coupling between the arm posture during the movement and the final arm posture was assessed by canonical correlation analysis evaluating the percentage of inter-trial variance of the final arm posture that could be explained by the variance of arm posture at a given sampling point. The canonical correlation analysis returns a *coefficient of determination*, which equals the mean *R*^2^ across the multiple regressions explaining the final arm posture as linear functions of the arm posture at a given sampling point.

#### Statistical analysis

Statistical analysis was calculated using SPSS 9.0. Pairwise comparisons were calculated for the temporal and spatial measures of the reaching movements, the percentage of total variance explained by the first two eigenvalues, as well as for the coefficient of determination. A repeated measurement ANOVA with condition (control vs. ischemia) as the between-group factor, and sampling point as the repeated factor was calculated for the following dependent variables: (1) standard deviation of arm posture, (2) standard deviation of hand position, and (3) standard deviation of hand orientation. Bonferroni corrected pairwise comparisons were calculated for *post-hoc* analysis of significant interactions. A Greenhouse-Geisser adjustment was made if the sphericity assumption was rejected by Mauchly's sphericity test. Standard deviations of arm posture, hand position and hand orientation were tested for normal distribution with the Lilliefors-test. Data was normally distributed for both conditions and for almost all sampling points. The critical value for significance was set at *p* < 0.05. Participants were excluded from single analyses in case of data corruption, i.e., if the data matrix for each participant contained <10 valid trials in each condition and for each target position.

## Results

Since the influence of target position on complex reaching movements was not of interest in the current study, and was already discussed elsewhere (Krüger et al., 2011, 2012), only the results for reaching toward the left target position will be presented here. Similar results were found for reaching movements toward the right target position, though in general the observed differences were smaller for the right target position as compared to the left target position.

### Temporal movement characteristics

**Figure 2:**
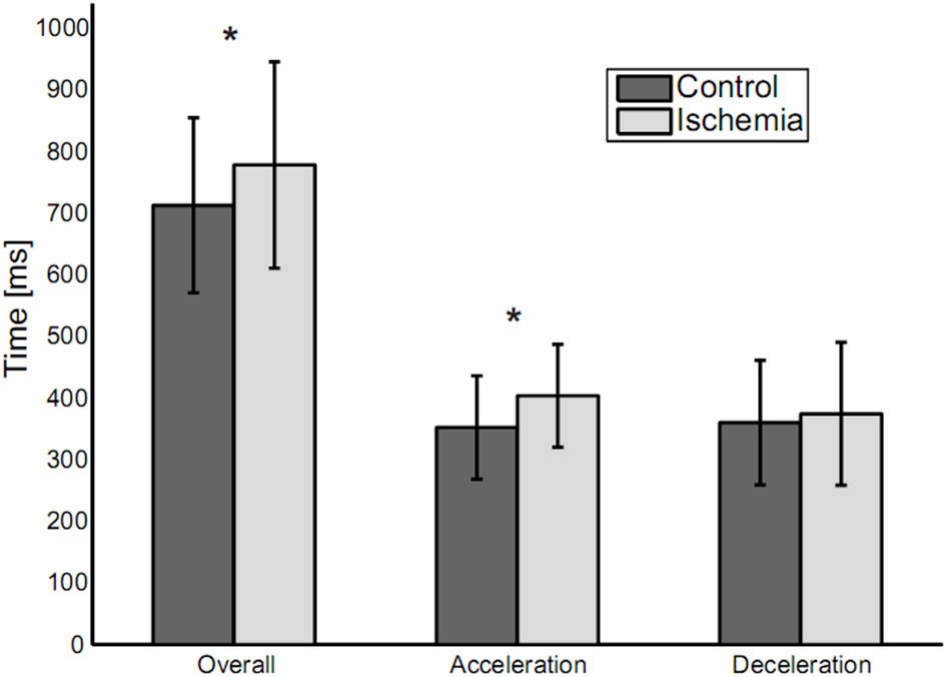
Movement durations (means ± standard deviation) for the three analyzed parameters: Overall movement duration, duration of acceleration and duration of deceleration. Statistically significant differences between experimental conditions are indicated by an asterisk.

Overall movement duration was 778 ± 167 ms (mean ± SD) for the ischemia condition and 713 ± 142 ms for the control condition (see Figure 2). This difference was significant [paired *t*-test: *t*_(14)_ = −3.55, *p* < 0.01] and based on a significantly increased duration of the acceleration phase under ischemia [403 ± 83 vs. 352 ± 84 ms, *t*_(14)_ = −3.08, *p* < 0.01]. Neither duration of the deceleration phase (375 ± 116 vs. 360 ± 101 ms), nor peak velocity (1,068 ± 198.57 vs. 1,095 ± 195 mm/s) differed between the ischemia and control condition.

### Spatial movement characteristics

**Figure 3:**
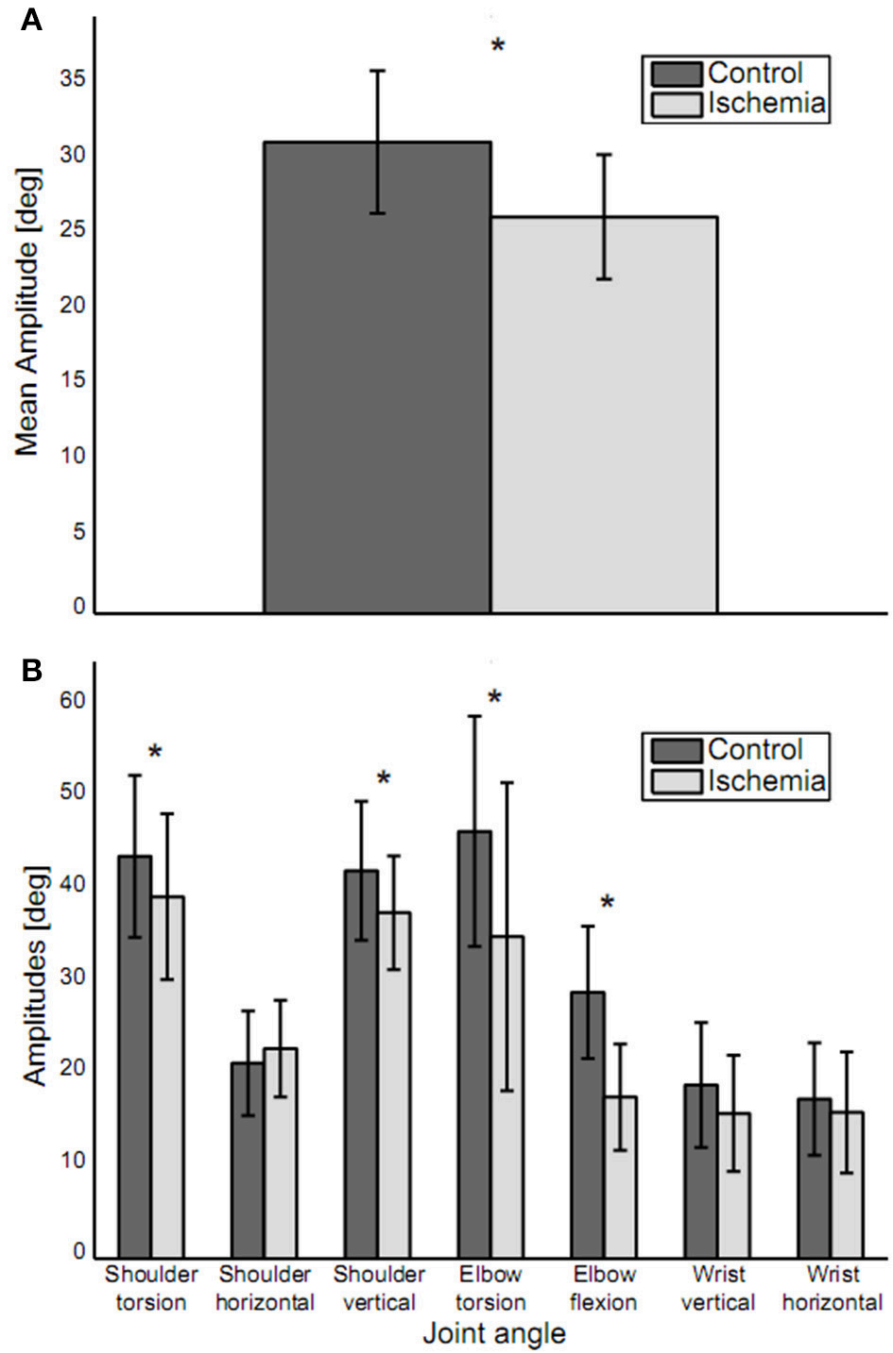
Movement amplitudes. **(A)** Mean movement amplitude (mean ± standard deviation) is depicted. Movement amplitude was significantly decreased under ischemia. **(B)** Movement amplitudes for each joint (mean ± standard deviation). Asterisks indicate significant differences between the two conditions. Movement amplitude was decreased in joints distal, as well as proximal to the blood pressure cuff.

When reaching toward the target, trajectories for five out of the seven joint angles of the arm (shoulder torsion, shoulder horizontal, shoulder vertical, elbow torsion, and wrist horizontal) showed a continuous increase or decrease between movement initiation and movement end, with the trajectories slightly curved. For elbow flexion and wrist vertical, joint angle trajectories showed a reversal in movement direction during the movement. Under ischemia, total path length in the 7D-joint space was decreased by 15% (control: 40.8 ± 6.8° vs. ischemia: 34.3 ± 5.4°). Associated with that, the participants' mean movement amplitude decreased significantly under ischemia as compared to the control condition [26.3 ± 4.1° vs. 31.3 ± 4.7°, *t*_(14)_ = 5.32, *p* < 0.01, see Figure 3A]. This difference was especially pronounced in four of the seven joint angles: shoulder torsion [*t*_(14)_ = 2.46, *p* = 0.03], shoulder vertical [*t*_(14)_ = 2.95, *p* = 0.01], elbow torsion [*t*_(__14)_ = 3.93, *p* < 0.01], and elbow flexion [*t*_(14)_ = 5.50, *p* < 0.01, see Figure 3B].

### Movement variability

**Figure 4:**
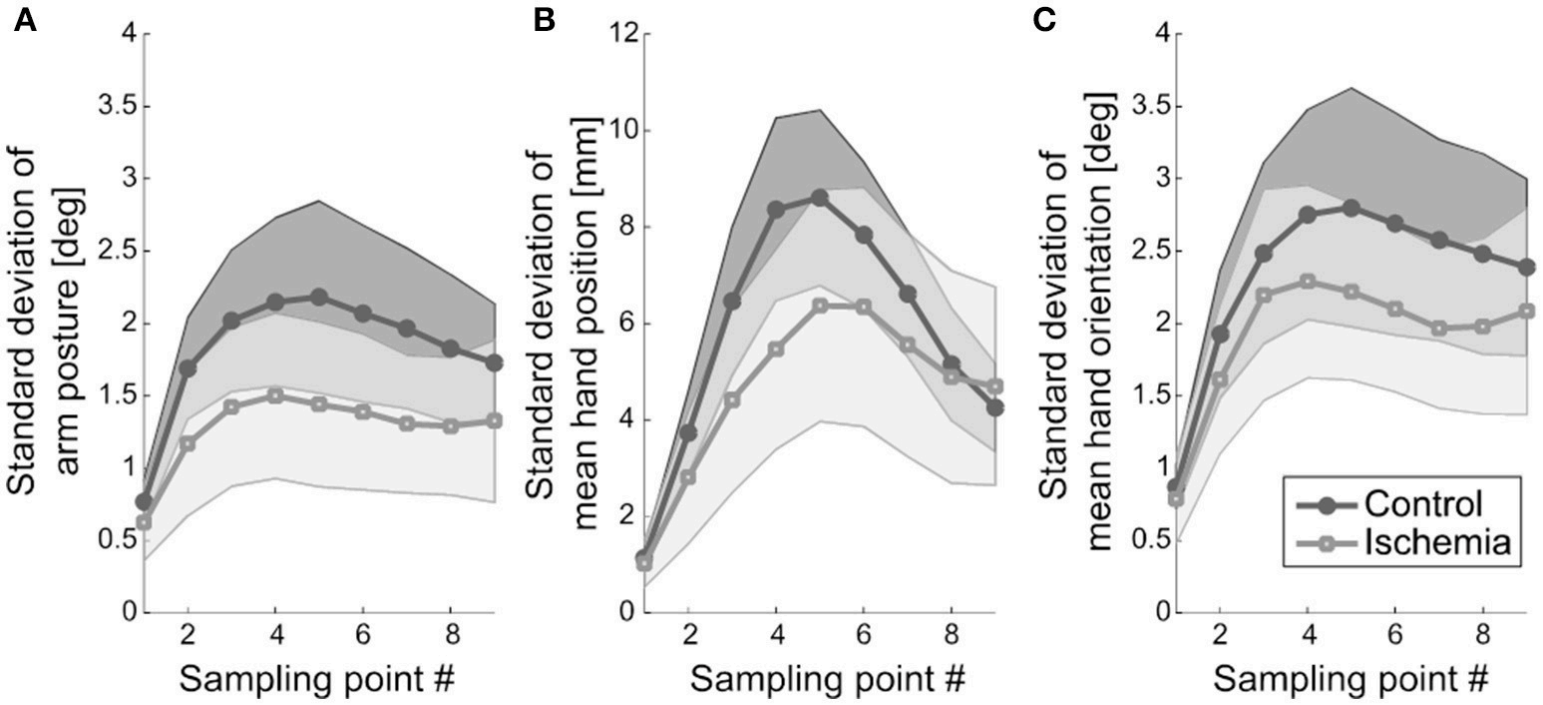
Movement variability. **(A)** Standard deviation of arm posture, representing the mean across participants, is shown. Shaded areas represent the respective confidence intervals. **(B)** Standard deviation of mean hand position (+ confidence interval) is depicted. Hand position variability was less modulated under ischemia. **(C)** Standard deviation of mean hand orientation and the respective confidence interval is shown.

First, movement variability at the different sampling points during movement execution was analyzed with respect to three measures: (a) standard deviation of arm posture, (b) standard deviation of hand position, and (c) standard deviation of hand orientation. The amount of movement variability did not differ between the two experimental conditions (i.e., no significant main effect of experimental condition) for any of the three measures neither across the nine sampling points nor at movement end. However, for each of the three measures, a significant main effect of sampling point became evident: (a) *F*_(2.39, 23.92)_ = 21.21, *p* < 0.01, (b) *F*_(2.36, 23.62)_ = 53.35, *p* < 0.01, and (c) *F*_(2.48, 24.83)_ = 22.93, *p* < 0.01. In all cases, movement variability increased until the middle of the movement and decreased afterwards. Variability was smallest at movement initiation and on an intermediate level at movement end (see Figure 4).

The interaction of experimental condition × sampling point was significant for standard deviation of hand position. Qualitatively, this effect became evident as a weaker modulation of hand position variability across the nine sampling points in the ischemia condition (see Figure 4B). *Post-hoc* analysis revealed that, under ischemia, only the first two sampling points differed largely from the other sampling points, whereas in the control condition almost all sampling points differed significantly from each other (see Table 1). No other effects reached the level of significance.

**Table 1:** *Post-hoc* analysis for the significant interaction Condition × Sampling point for the measure: Standard deviation of hand position.

	**Sampling point no**.	**Ischemia**								
		**1**	**2**	**3**	**4**	**5**	**6**	**7**	**8**	**9**
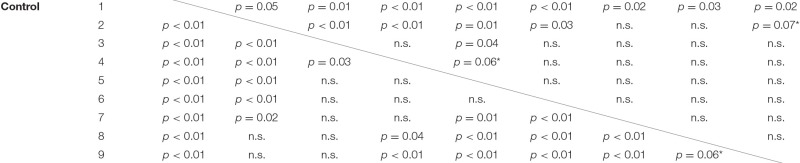										

*Data in the upper right half of the table represents p-values of significant pairwise comparisons of single sampling points for the ischemia-condition. Data in the lower left half of the table represents p-values of significant pairwise comparisons of single sampling points for the control condition. Bonferroni correction for multiple comparisons was applied to all calculations. Asterisks indicate close to significant interactions*.

**Figure 5:**
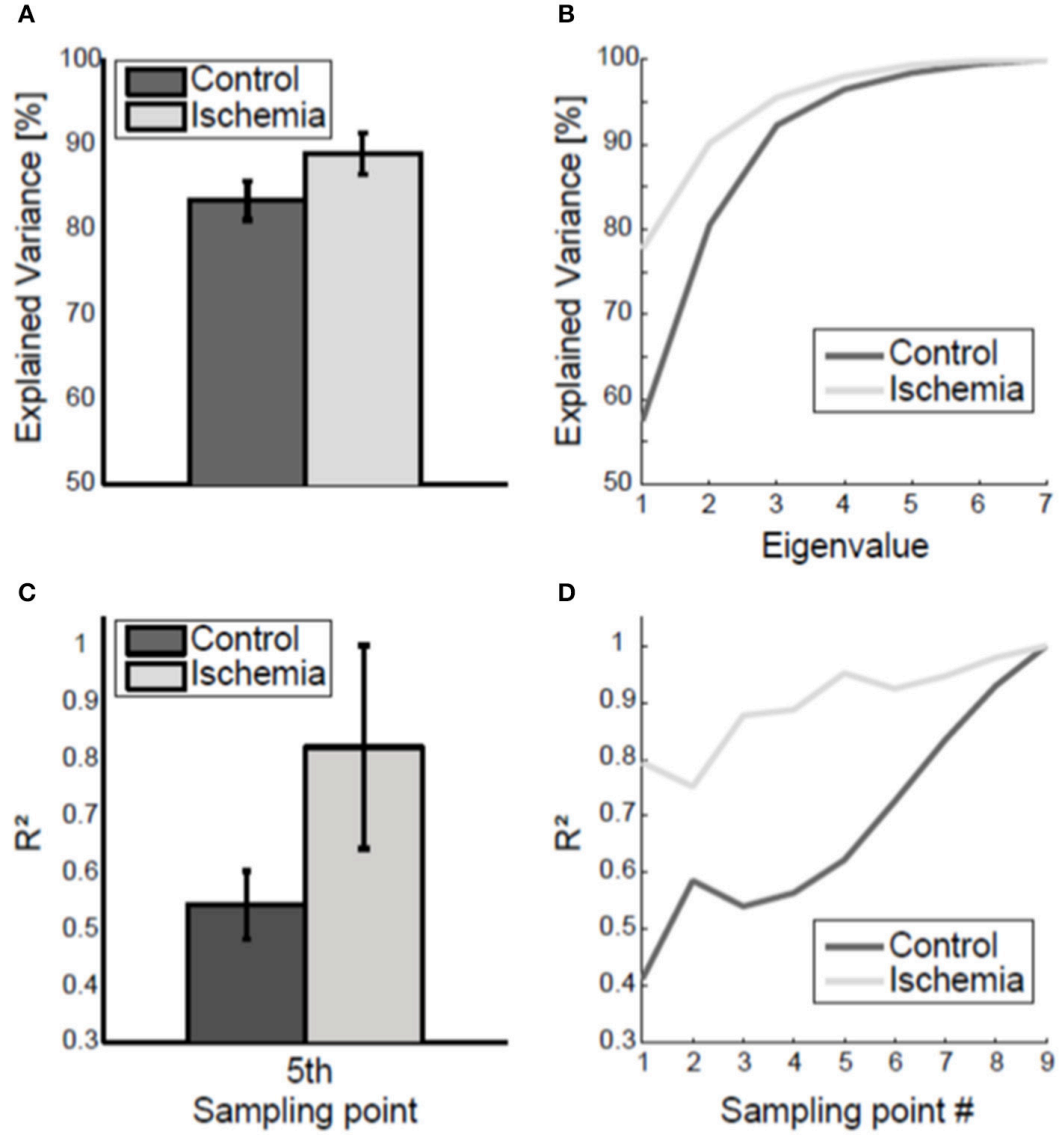
Coupling of joint angle variability. **(A)** Group mean (± standard deviation) of the variance explained by the two biggest eigenvalues, averaged across the nine sampling points, is shown. Under ischemia significantly more variance was explained by the first two eigenvalues as compared to the control condition. **(B)** Explained variance by the seven eigenvalues, averaged across the nine sampling points, is shown for one representative participant. **(C)** The coefficient of determination (*R*^2^) of final arm posture variance with respect to arm posture variance at the fifth sampling point. Error bars represent standard deviations. Under ischemia the coefficient of determination was higher than in the control condition. **(D)** The coefficient of determination (*R*^2^) of final arm posture variance with respect to arm posture variance for each sampling point is shown for a representative participant for both conditions.

The coupling of joint angles within the arm posture was analyzed using a principal component analysis applied to the inter-trial 7 × 7 covariance matrix of the arm posture. Under ischemia, the first two eigenvalues explained 88.90 ± 2.44% of total joint angle variance compared with 83.40 ± 2.27% in the control condition (see Figure 5A for group mean and Figure 5B for a representative participant). This difference was significant [*t*_(8)_ = −18.43, *p* < 0.01].

The coupling of the arm posture was analyzed using the coefficient of determination between of final arm posture with respect to the arm posture during movement execution. As a matter of course, the coefficient of determination increased toward movement end and finally reached the level of 1 (see Figure 5D for a representative participant). The coefficient of determination of final arm posture with respect to the variance of the arm posture at the first sampling point was smaller in the control condition (*R*^2^ = 0.4) than under ischemia (*R*^2^ = 0.7). Consequently, the subsequent increase in the coefficient of determination up to the value 1 at movement end was steeper in the control condition than under ischemia. For group comparison, only the coefficient of determination with respect to the fifth sampling point, when the standard deviation of arm posture was maximal, was analyzed. Under ischemia the coefficient of determination was significantly higher than in the control condition [*R*^2^: 0.82 ± 0.18 vs. 0.54 ± 0.06, *t*_(7)_ = −3.89, *p* < 0.01, see Figure 5C].

## Discussion

In the current study, we introduced a method to investigate the coupling of joint angle variability across the time course of discrete, goal-directed, natural reaching movements. The method was exemplified on a dataset that was collected to study the influence of temporary proprioceptive deafferentation on the control of a complex reaching movement. In the following, the main outcomes of the study will be discussed first, followed by a discussion on the canonical correlation method introduced to analyze the propagation of movement variability across time.

### Adjustment of movement duration

Movement duration was increased by the ischemia as a result of increased acceleration duration. The influence of proprioception on the duration of acceleration was already recognized by Bagesteiro et al. (2006) and associated with sensory-based online-correction of the movement. Movement's peak velocity was not increased under ischemia. Increased duration of acceleration without increased peak velocity indicates decreased peak acceleration and, consequently, decreased peak force. A reduction in total force applied during movement execution is accompanied by a reduction in signal-dependent noise (Harris and Wolpert, 1998). This may be advantageous under ischemia, as the precision of movement planning is of greater importance when movement online-control based on proprioceptive feedback is impaired. Our results suggest that healthy participants are able to immediately and efficiently adjust the precision of movement planning to the lack of proprioceptive information in elbow and wrist.

### Adjustment of movement amplitude

Movement amplitude was decreased under ischemia. Importantly, this was not only true for joints distal to the applied blood pressure cuff (i.e., elbow torsion and elbow flexion), which were directly affected by the ischemic block, but also for two joint angles proximal to the cuff (i.e., shoulder torsion and shoulder vertical), which were not directly affected by the ischemia. In combination with the finding of stronger inter-joint coupling under ischemia, this suggests a more global change in the strategy of joint angle coordination involving all joints of the arm to compensate for the ischemia. A reason for planning a reaching movement with decreased mean movement amplitude may be the associated decrease in the signal-to-noise ratio (Harris and Wolpert, 1998), facilitating the control of movement endpoint variability. This assumption is also supported by Fitts' Law (Fitts, 1954), which describes the relationship between movement amplitude, movement duration and movement accuracy. According to this law, in order to keep movement endpoint variability constant in a task with increased task difficulty, movement duration and/or movement amplitude must be adjusted. Assuming that the ischemia may have increased task difficulty, as an important source of sensory information was disabled, planning a movement with decreased movement amplitude and increased movement duration may have allowed the participants to keep movement endpoint variability constant, as observed in our study.

### Adjustment of movement variability

Another important finding of our study was that the modulation of hand position variability during movement execution was altered under ischemia in such a way that the initial increase and subsequent decrease of hand position variability was less pronounced. The increase-decrease pattern of movement variability was already described in earlier studies by our group (Krüger et al., 2011, 2012) and is a sign of successful minimization of variance at movement end. It indicates that signal-dependent noise (Harris and Wolpert, 1998), introduced by forces during the acceleration period, is successfully compensated by feedback control acting primarily during the deceleration phase (Elliott et al., 2001, 2010; Eggert et al., 2016). The fact that this increase-decrease pattern of hand position variability is less pronounced under ischemia (see Figure 4) is probably related to both reduced acceleration forces, resulting in a reduced increase of variability, and impaired proprioceptive feedback, resulting in a reduced decrease of variability. Interestingly, both of these changes compensated for each other in such a way that endpoint variability was almost identical in the control condition and under ischemia. This is in contrast to findings of studies with chronically deafferented patients (Gentilucci et al., 1994; Gordon et al., 1995; Nougier et al., 1996; Medina et al., 2009) and reflects the ability of the motor control system of healthy participants to immediately and efficiently adjust to the loss of proprioceptive information in parts of the effector.

### Adjustments of movement coordination

Movement coordination was altered under ischemia, a finding similar to that observed in studies on deafferented patients (Sainburg et al., 1993; Ghez and Sainburg, 1995; Sarlegna et al., 2006). In the current study, the alterations in movement coordination became evident for the coupling between single joints of a specific arm posture as well as for the coupling of arm posture during movement execution with that at movement end: for both parameters the coupling was stronger under ischemia. Increasing the strength of joint angle coupling under ischemia, i.e., increasing the synergistic coordination of the redundant DoF at the same time point and across time points, may reflect a change in the control strategy concerning the way motor redundancy is used under impaired proprioceptive feedback. Alternatively, it may reflect the limited capacity of the brain to plan and control coordinated movement with a naturally higher number of DoF with decreased proprioceptive feedback. Similar to that assumption, Gupta (2014) highlighted the relevance of feedback information for the precise temporal control of complex motor actions. Independent of which explanation holds true, the observed effect of stronger joint angle coupling under ischemia can be interpreted as a reduction of the number of kinematic DoF of the redundant effector-system arm and consequently as a facilitation of its online-control.

### Methodological considerations

In this study, canonical correlation analysis was introduced as a method to investigate the propagation of movement variability for discrete movements involving a redundant effector system. It was shown that, by using canonical correlation analysis, alterations in movement coordination that indicate changes in the control strategy concerning the use of motor redundancy could be detected. To the best of our knowledge, this is the first time such an approach is suggested for a multiple DoF-effector system in the context of discrete movements. Previous approaches to capture the propagation of movement variability for discrete movements have studied either eye movements (West et al., 2009; Richardson et al., 2011; Eggert et al., 2016) or arm movements with a limited number of DoF (Messier and Kalaska, 1999; Heath, 2005; Kuang and Gail, 2015). However, to advance our understanding of the human motor control system in its complexity, natural movements as used in this study have to be analyzed. In this context, the introduction of canonical correlation analysis as a suitable approach represents an important methodical advance in the context of neuromechanics. One aspect contributing to the importance of the introduced method is that it does not substitute or extend other approaches that have been previously suggested to identify structure in movement variability, but represents a new approach with the potential to also broaden the application of already existing methods and to increase their significance: In this study, it was analyzed how strongly final arm posture variability was determined by overall variability of arm posture during movement execution. Previous studies identified different components of overall movement variability (Müller and Sternad, 2004, 2009) and the relevance of independent components of effector variability for task variability (Scholz and Schöner, 1999; Latash et al., 2002). Following this line of thinking, future research could use canonical correlation analysis to investigate the propagation of certain components of overall effector variability in time.

## Conclusions

In this study, we introduced canonical correlation analysis as a method to investigate the temporal propagation of movement variability in reaching movements under normal or impaired proprioceptive feedback. As general findings, we found increased movement duration due to increased acceleration duration, decreased movement amplitude, as well as changes in movement coordination under reduced proprioceptive afference due to ischemia. The changes in movement coordination became evident as an increased coupling between arm postures during movement execution with final arm posture, resulting in a decreased number of kinematic DoF of the effector-system. Movement endpoint variability was not influenced by the ischemia. Thus, the canonical correlation analysis revealed that healthy participants are able to immediately and efficiently adjust their control strategy to the impaired flow of proprioceptive information. In conclusion, canonical correlation analysis provides a valuable method to advance our understanding of human movement control by offering an approach to analyze the temporal propagation of movement variability during discrete movements executed by multiple DoF-effector systems, such as the arm.

## Author contributions

All authors of this study contributed substantially to its conception, the interpretation of data, as well as drafting and revising earlier versions of the manuscript. In addition, MK and TE were responsible for data acquisition and analysis. MK, AS, and TE all approved the version to be published and agreed to be accountable for all aspects of the work.

### Conflict of interest statement

The authors declare that the research was conducted in the absence of any commercial or financial relationships that could be construed as a potential conflict of interest.
